# Homo- and Heterotypic Trajectories in a Preschool to Primary-School Clinical Sample: A Prospective Study Related to Maternal Psychopathology

**DOI:** 10.3389/fpsyt.2019.00153

**Published:** 2019-03-22

**Authors:** Isabell Jobs, Jörg Michael Müller, Olena Skorozhenina, Georg Romer

**Affiliations:** ^1^Department of Child and Adolescent Psychiatry, University Hospital Münster, Münster, Germany; ^2^Health Psychology and Applied Diagnostics, University of Wuppertal, Münster, Germany

**Keywords:** homotypic, heterotypic development, comorbidity, preschool, school age, clinical sample, maternal psychopathology

## Abstract

**Background:** Most longitudinal or follow-up mental health studies describe developmental pathways using dimensional measures of psychopathology, but seldom using pathways described by clinical disorders.

**Objective:** We aim to describe diagnostic pathways by homotypic (within the disorder continuity) and heterotypic development (between the disorder continuity), with maternal psychopathology as moderator for both trajectories.

**Methods:** Clinically referred children (0–7 years; *N* = 83) were assessed at preschool age and at primary-school age through a clinical interview. We built a disorder cluster of emotional disorders (ED; F32, F40, F42, F43, F93.0, F93.1, F93.2, F93.8, F95), behavioral disorders (BD; F68.8, F90, F91, F91.3, F93.3, F93.9, F94), and specific early onset disorders (SEO; F50, F51, F70, F98.0, F98.1, F98.2, F98.8, F98.9). We describe the prevalence, comorbidity, and clinical trajectories of various types of homotypic and heterotypic development.

**Results:** We observed a high rate of comorbidity throughout the study (62.6% at admission and 67.5% at follow-up) and in general, a high continuity of mental health problems from preschool to primary-school age children (69.9% of the sample showed continuity), with 50.6% of the sample showing homotypic and 44.6% showing heterotypic development. Hierarchical multiple regression analyses suggest that heterotypic development may be influenced by maternal psychopathology.

**Conclusion:** Currently, evidence-based mental health guidelines for preschool populations are designed and evaluated assuming a homotypic development. However, our findings indicate that treatment interventions and outcome measures should also be designed and evaluated for heterotypic development especially in case of increased maternal psychopathology.

## Introduction

Developmental changes in preschool-age children produce a great variation in behavior and emotional states and, for a subpopulation with an exaggerated symptom level, these problems do not seem to be transient [e.g., (1)]. In order to comprehend clinical pathways and their development in the very young years, it is important to distinguish between homotypic and heterotypic development and symptom comorbidities [see e.g., (2, 3)]. The homotypic pathway describes the continuity of a particular mental health problem over time and this pathway is widely assumed to be at play in most empirical clinical studies; conversely, the heterotypic pathway describes the development of a new clinical disorder. Both pathways have been recently examined in several studies (2–4) and can shed light on disease etiology and help shape treatment planning. For example, (4) described homotypic development for anxiety, ADHD, and disruptive behavior disorder in a community sample (*N* > 500 from preschool to middle childhood). Such homotypic development starting in preschool age is described frequently by broader disorder categories, such as behavioral disorders (abbreviated BD or sometimes labeled as externalizing behavior) and emotional disorders (abbreviated ED or sometimes labeled as internalizing behavior).

Nevertheless, irrespective of homotypic development, children may undergo (additional) heterotypic development, which points to the possibility of a shared etiology mechanism (e.g., environmental, such as parents' and/or mother's psychopathology or genetics), or a network interaction between disorder clusters [see (2, 5)]. An example of a network interaction is the failure model by (6), which indicates that the negative feedback caused by externalizing behavior can have the long-term effect of turning into internalizing or depressive symptoms (BD-ED); such a clinical pathway was also described by (7). However, (7) described a negative predictive path from early internalizing problems to externalizing problems (ED-not BD), which means that children with early internalizing symptoms showed underproportional (less) externalizing behavior later on, such that there was not heterotypic development.

### The Challenge of Describing Clinical Trajectories Based on Categorical Measures

The view of mental disorders as fixed and independent entities (2) is in agreement with a homotypic development, which implies different etiological factors. If we assume that an etiological factor may cause a number of different mental disorders (by a certain probability), then we will more frequently observe comorbidity or a heterotypic development than predicted by a single mental disorder. Nevertheless, comorbidity does not necessarily indicate shared etiological factors. Therefore, the first methodological challenge for distinguishing between homotypic and heterotypic development is to exclude the comorbidity of clinical disorders as an explanation. As such, large community samples are needed to achieve sufficient statistical power to predict the occurrence of disorders with low frequencies in a community sample in order to disentangle the predictive power of single variables. One example that met this requirement was the study by (2), which provided evidence for homotypic and heterotypic development of mental health disorders for a large adult sample (18–64 years; >50.000). Later, (3) replicated Lahey et al.'s results for a community sample of children (Alspac study; *N* > 1.500 children from 7.5 to 14 years), but that study was based on quasi-diagnoses (derived from dimensional measures of psychopathology, especially from externalizing problems, such as ADHD, to internalizing disorders). Additional methodological challenges for examining heterotypic development, particularly in samples of children at preschool age, are the uncertainties surrounding clinical diagnoses made by using recently developed clinical interviews [e.g., PAPA from (8)], biases in maternal reports (9), and the higher base rates of normative variation for single clinical symptoms [see (10)] at preschool age.

In order to reduce the influence of comorbidity and diagnostic uncertainty, some studies have examined clinical trajectories in broader clinical terms, such as disorders of distress, fear, or externalizing disorders [see e.g., (2, 3)], or in terms of externalizing (EXT) and internalizing (INT) problem behavior. Authors such as (11) used the 28 different DSM-III-R diagnoses to define broader disorder clusters, namely e*motional disorders* (ED; including simple phobia, dream anxiety disorder, separation anxiety disorder, overanxious disorder, avoidant disorder, anxiety disorder not otherwise specified, depressive disorder, dysthymia, posttraumatic stress disorder, and adjustment disorders), *disruptive disorders* (DD; including oppositional defiant disorder, attention deficit disorder, and conduct disorder), and *not otherwise specified* (NOS; including sleepwalking-sleep talking, functional enuresis, and encopresis).

To our knowledge, no study has yet examined maternal psychopathology as a condition for homotypic and heterotypic development. Two influences for homotypic and heterotypic development seem reasonable to us: First, we can conceptualize maternal psychopathology as a model for the child [see e.g., (12)]. This conception contains multifold aspects. For example, maternal psychopathology may guide a child's awareness of their own or others' emotions, expressions of emotion or behavior related to emotions; it may guide how children organize their daily activities; or children may use the mother–child interaction as model for social interactions [for different emotion-regulation strategies, see (13)]. We do not know which aspect might predict a more homotypic development, but if maternal psychopathology triggers the development of a specific disorder (e.g., ED or BD), this continuing condition will cause a homotypic development (depending on the temperamentality or vulnerability profile of the child). Second, we conceptualize maternal psychopathology as an external stressor for the child [e.g., lower quality in parenting behavior, see (14); and negative maternal behavior and disengagement from the child, see (15)], possibly causing any type of unspecific or mental disorder, depending on the temperamental or vulnerability profile of the child, which will probably predict a more heterotypic development. Both mechanisms may have an impact, especially for preschool-aged children, because of greater normative changes and variability in development at this age (10).

Aside from such maternal influences, the type of mental health problem, such as emotional or behavioral disorders as noted above, may also trigger homotypic or heterotypic development. In particular, diagnoses such as enuresis or encopresis may represent unspecific precursors to clinical disorder clusters (16, 17); we cover this aspect later under the term ‘specific early onset disorders' (see below).

## Aim of the Study

We aim to estimate the influence of maternal psychopathology on homotypic and heterotypic development across clusters of disorders, namely *behavioral disorders* (BD; externalizing), *emotional disorders* (ED; internalizing), and *specific early onset disorders* (SEO). These analyses are based on the descriptions of prevalence, comorbidity, and clinical trajectories in a clinical sample. We aim to describe the base rates of the broad diagnostic categories (BD, ED, SEO) and their comorbidities in a clinical sample of children at preschool age and at primary-school age. Furthermore, we describe all combinations of diagnostic categories in order to illustrate clinical pathways described by homo- and heterotypic development. Finally, we examine the influence of maternal psychopathology (assessed at admission, end of day treatment, and at follow-up) for homo- and heterotypic development.

## Methods

### Procedures

This longitudinal cohort study was approved by the local ethics committee of the University Hospital Muenster. The inpatient sample (mothers and their children) was clinically evaluated in the Family Day Hospital in Münster, a city in Northern Germany (approximately 307,000 inhabitants in 2016), from 2001 to 2012. The intervention [for details see (18)] comprised behavioral (video feedback of the parent–child interaction) and psychodynamic techniques for parents (addressing the maternal mental representations of the child) and functional therapies (psychomotor treatment) for children. After discharge 51.3% of the children received a subsequent therapeutic intervention within the follow-up period (*N* = 7 missing), while subsequent maternal treatment was not assessed. The probability of children from mothers with a GSI above the cut-off for an intervention in the follow-up period is not significant (Chi-Square = 0.16; df = 1, *p* = 0.69). From all mother-child dyads with a nearly complete clinical record (related to questionnaires, sociodemographic data from admission, and discharge documentation), 255 were contacted by telephone between 2007 and 2016 to participate in a follow-up study [see (18)] and 97 filled out the informed consent form. The reasons for non-participation, aside from non-contact, were mainly “effort/moved away” (42.4%), no time, disappointment with the therapeutic intervention, or disinterest (41.1%). We excluded two patients with inconsistent clinical records related to diagnosis. The final sample consisted of 83 participants, who were interviewed for 60 to 90 min with the *Diagnostisches Interview bei psychischen Störungen im Kinder- und Jugendalter* (19, 20) within meeting rooms of the Child and Adolescent Psychiatry Unit of the University Hospital Muenster. Additionally, parents filled out questionnaires (see below) and received an expense allowance (voucher of about 40€).

#### Sample

##### Children

At the time of admission, the average age of the children was 4.5 years (min = 0.33; max = 7.0 years) and 8.8 years (min = 3.4; max = 14.1 years) at follow-up. The sample consisted of 75.9% boys and 24.1% girls. Most of the children had at least one sibling (28.4 % without sibling). The majority of the children (85.2%) lived with both parents.

##### Mothers

At admission, the average age of the mothers was 33.3 years (age range: 23–43). Most of the mothers were German (96.3 %). Of the participating mothers, 43.1 % completed an intermediate school certificate and 21.5 % completed their A-levels (12–13 years of school). At the point of admission, 80.8 % were married, 5.1% divorced, and 10.3 % single. At follow-up, 71.7 % were married, 11.5 % were separated, 5.1% divorced, and 10.3 % single.

#### Measures

##### Assessment of Diagnoses

Clinical records comprise the database for the first assessment at the beginning of treatment at preschool age. At primary-school age, parents or caretakers were interviewed with the *Diagnostisches Interview bei psychischen Störungen im Kinder- und Jugendalter* (19, 20) designed for children and adolescents. The first part of the interview was a screening portion to evaluate problems over the last 6 months and the second part evaluated specific diseases typical in early and later childhood. Using fixed predefined questions with defined and free answers, the following topics were covered: attention deficit, activity and social behavior, tic disorder, anxiety disorder, encopresis and enuresis, sleep disturbance, affective disorder, and eating disorder. Finally, the interview evaluated substance abuse and detailed psychiatric and family anamnesis. Diagnosis and classification of diseases were made by DSM-IV-TR and ICD-10-Diagnosis. Retest-reliability and validity were verified (19, 20).

##### Disorder Cluster

We grouped single diagnoses into broader disorder clusters following the study by (11), with minor deviations. In detail, we developed the *emotional disorder cluster (ED, internalizing disorders)* to contain the diagnoses F32, F40, F42, F43, F93.0, F93.1, F93.2, F93.8, and F95, and the *behavioral disorder cluster (BD, externalizing cluster)* contained F68.8, F90, F91, F91.3, F93.3, F93.9, and F94. Similar to Lavigne et al., we additionally created a separate cluster for remaining diagnoses, but we labeled them according to their content as *specific early onset disorders (SEO)*, containing F50, F51, F70, F98.0, F98.1, F98.2, F98.8, and F98.9.

This method of creating disorder clusters has numerous advantages such as sufficient cell frequencies, simplified analyses, and increased reliability via aggregation. Moreover, our results can be compared to those from dimensional assessment approaches that also assess broader diagnostic categories with broadband externalizing and internalizing scales.

##### Defining Homotypic and Heterotypic Development

**Figure 1:**
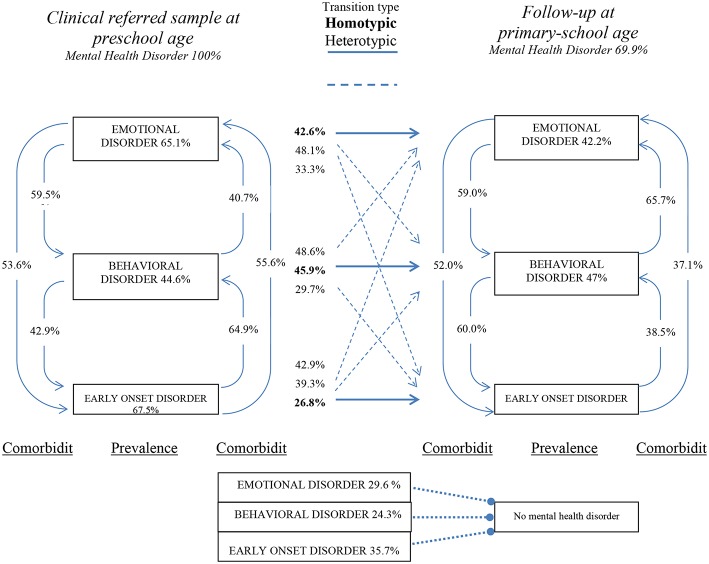
*Percentage* of children showing a disorder in one of three disorder clusters (ED, BD, SEO) at admission and follow-up, *comorbidities* between disorder clusters at preschool (left side) and primary-school age (right side), and *transition types* (center: homotypic or heterotypic). *N* = 83. Any heterotypic development is indicated by a dashed line, while a solid line indicates homotypic development.

We define *homotypic* and *heterotypic development* as a specific combination of our disorder clusters defined above. Children who stayed within one disorder cluster (e.g., *ED* at preschool age and *ED* at primary-school age) showed homotypic development (continuity of a particular mental health problem over time) and children showing a new disorder (e.g., only *ED* at preschool age and *ED* and *BD* at primary-school age) showed heterotypic development. A detailed description of all combinations is presented in Figure 1 based on42 children with homotypic development and 37 with heterotypic development.

#### SCL-90-R

Mothers were asked to complete the German version of the Symptom Checklist-90-R (21) at admission, discharge, and follow-up, using a 5-point scale (0 = “no problem” to 4 = “very serious”) to answer questions about maternal psychopathology. The SCL-90-R is a well-established instrument with proven reliability and validity (22). The questionnaire includes 90 items for evaluating the last seven days in self-report. This study also used the GSI (Global Severity Index) and defines a clinical cut-off score of ≥0.77, such that values higher than the cut-off are clinically relevant. At admission, the GSI score was abbreviated as MPP1 (for maternal psychopathology), at discharge as MPP2, and at follow-up as MPP3, and the percentage of mothers with a GSI score above the cut-off score was 56.6%, 20.5%, and 24.1%, respectively (rMPP1-2 = 0.63^**^; rMPP1-3 = 0.65^**^; rMPP2-3 = 0.42^**^). The GSI-score form the SCL-90-R was associated with the BDI with *r* = 0.74^***^ [see (23)].

#### Statistical Analyses

The prevalence rates for the disorder clusters, comorbidities, and homotypic or heterotypic development are indicated by percentages, odds ratios, and cross tables, respectively. Additionally, the Statistical Package for Social Sciences (SPSS for Windows, SPSS 25, IBM Corporation) was used to obtain multiple logistic regressions to examine predictors for homotypic or heterotypic development. Age and assessment intervals were scaled in months within the logistic regression analysis.

#### Handling Missing Data

From our initial sample of *N* = 255 we have an initial GSI-score for *N* = 108 which did not take part at follow up. This offers the opportunity to check for a systematic drop out. We tested for systematic drop-out by comparing the GSI score at admission between the sample with (*N* = 83) and without (*N* = 108) a GSI score at follow-up and the *t*-test for independent groups showed no significance difference (*t* = 0.94, df = 167) in the mean scores. In order to avoid a loss of available data through single occurrences of missing data in the multivariate analysis, we performed multiple (*n* = 10) imputation.

## Results

Table 1 shows the percentage of children presenting at least one disorder within a disorder cluster (BD, ED, and SEO) at preschool and at primary-school age and Table 2 shows the odds ratios for comorbidity at preschool age and at primary-school age.

**Table 1:** Frequencies of single disorders and emotional disorders cluster (ED), behavioral disorders (BD), and specific early onset disorders cluster (SEO) at preschool age and primary-school age (*N* = 83) in percentages of ICD-10 diagnoses; multiple disorders for each preschool child were possible.

		**Clinical sample at preschool age *%***	**Follow-up at primary-school age *%***
**ICD-10 Code**	**Diagnosis**		
	**Behavioral Disorders (BD) F68.8, F90, F91, F91.3, F93.3, F93.9, F94**	**44.6**	**47.0**
F68.8	Other specified disorders of adult personality and behavior	14.5	–
F90	Hyperkinetic disorder	16.9	31.3
F91	Conduct disorders	1.2	6.0
F91.3	Conduct disorders, oppositional defiant disorder	–	37.3
F93.3	Sibling rivalry disorder	4.8	–
F93.9	Childhood emotional disorders, unspecified	–	–
F94^*^	Disorders of social functioning with onset specific to childhood and adolescence	7.2	18.8^*^
	**Emotional Disorders (ED) F32, F40, F42, F43, F93.0, F93.1, F93.2, F93.8, F95**	**65.1**	**42.2**
F32	Depressive episode	–	2.4
F40	Phobic anxiety disorders	–	24.1
F42	Obsessive-compulsive disorder	–	2.4
F43	Reaction to severe stress, adjustment disorders	1.2	1.2
F93.0	Separation anxiety disorder of childhood	3.6	10.8
F93.1	Phobic anxiety disorder of childhood	–	2.4
F93.2	Social anxiety disorder of childhood	6.0	10.8
F93.8	Other childhood emotional disorders	56.6	4.8
F95^*^	Tic disorders	–	21.9^*^
	**Specific early onset disorders (SEO) F50, F51, F70, F98.0, F98.1, F98.2, F98.8, F98.9**	**67.5**	**30.1**
F50	Eating disorder	6.0	–
F51	Non-organic sleep disorders	13.3	34.4^*^
F70	Mild intellectual disabilities	2.4	–
F98.0	Non-organic enuresis	10.8	13.3
F98.1	Nonorganic encopresis	12.0	–
F98.2	Feeding disorder of infancy and childhood	8.4	–
F98.8	Other specified behavioral and emotional disorders with onset usually occurring in childhood and adolescence (crying)	8.4	–
F98.9	Unspecified behavioral and emotional disorders with onset usually occurring in childhood and adolescence	2.4	–

* *Diagnoses are based on a subsample of 32 persons. Percentages in column and rows are adjusted*.

**Table 2:** Odds ratios of comorbidities of BD (behavioral disorder), ED (emotional disorder), and SEO (specific early onset) disorder clusters (see text) at preschool age and primary-school age (*N* = 83).

	**Criterion:comorbid disorder cluster**		
	BD	ED	SEO
**Predictor**	**Comorbidity at preschool**		
BD cluster	–	1.17	1.07
ED cluster	1.27	–	1.61
SEO cluster	1.12	1.66	–
	**Comorbidity at primary-school age**		
BD cluster	–	0.46^*^	0.59
ED cluster	0.51^*^	–	0.67
SEO cluster	0.69	0.73	–

* *p < 0.05*.

Figure 1 reports the percentage of children presenting at least one disorder within a disorder cluster at preschool (left side) and primary-school age (right side) together with the percentage of children who show a presence of a disorder at preschool age and primary-school age (labeled as transition types in the center) and partially illustrates information from Tables 1, 2 in a concise way, allowing a descriptive comparison of percentages. Figure 1 also illustrates the percentage of children who demonstrate heterotypic development, for example, ED at preschool age and ED plus BD at primary-school age. Moreover, Figure 1 contains percentages of comorbidity between the disorder clusters at each assessment, which facilitates comparisons of percentages in a descriptive way.

Table 3 shows a hierarchical multiple regression analysis to predict, in the first step, the homo- and heterotypic clinical trajectories depending on clinical cluster (ED, BD, SEO), age, and the assessment interval (time from discharge to follow-up assessment). In the second step, we add maternal psychopathology as a moderator variable at the point of admission, discharge, and follow-up (MPP1-MPP3).

**Table 3:** Hierarchical multiple logistic regression to predict homo- and heterotypic clinical trajectories depending on clinical disorder cluster (ED, BD, SEO; see text), sociodemographic variables (age, assessment interval), and maternal psychopathology as a moderator variable.

		**Estimate**	**SE**	**Odds ratio**	**95% CI**	***p***
**Overall Homotypic**	**Step 1**					
Chi^2^ = 22.04^***^	ED	0.99	0.58	2.69	0.86–8.39	0.089
Nagelkerkes *R*^2^ = 0.31	BD	1.99	0.56	7.35	2.44–22.22	0.000
	SEO	0.17	0.56	1.19	0.40–3.57	0.757
	Age T1	0.07	0.17	1.07	0.76–1.50	0.689
	Assessment Interval	0.02	0.01	1.03	1.00–1.05	0.062
	**Step 2**					
Chi^2^ = 4.72^***^	ED	1.20	0.63	3.32	0.96–11.50	0.058
Nagelkerkes *R*^2^ = 0.37	BD	−1.98	0.62	7.25	2.17–24.39	0.001
	SEO	−0.24	0.60	1.27	0.39–4.13	0.691
	Age T1	0.12	0.19	1.13	0.78–1.63	0.530
	Assessment Interval	0.03	0.01	1.03	1.00–1.06	0.033
	MPP1	−0.37	0.71	0.69	0.17–2.84	0.609
	MPP2	0.73	0.63	2.07	0.60–7.14	0.252
	MPP3	1.10	0.70	2.99	0.76–11.74	0.116
**Overall Heterotypic**	**Step 1**					
Chi^2^ = 23.33^***^	ED	−1.20	0.60	0.30	0.09–0.97	0.044
Nagelkerkes *R*^2^ = 0.33	BD	−1.68	0.59	0.19	0.06–0.59	0.004
	SEO	−2.04	0.64	0.13	0.04–0.45	0.001
	Age T1	−0.37	0.19	0.69	0.48–1.00	0.048
	Assessment Interval	0.01	0.01	1.01	0.98–1.03	0.580
Chi^2^ = 8.05	**Step 2**					
Nagelkerkes R^2^ = 0.42	ED	−1.22	0.64	0.30	0.08–1.03	0.056
	BD	1.72	0.66	0.18	0.05–0.65	0.009
	SEO	2.24	0.70	0.11	0.03–0.42	0.001
	Age T1	−0.49	0.22	0.61	0.40–0.94	0.024
	Assessment Interval	0.01	0.01	1.01	0.98–1.04	0.486
	MPP1	−1.14	0.76	0.32	0.07–1.44	0.138
	MPP2	1.89	0.76	6.60	1.49–29.29	0.013
	MPP3	−0.33	0.70	0.72	0.18–2.80	0.632

*** *p < 0.001*.

## Discussion

Our prospective study on a clinical sample of preschool-aged children examined maternal psychopathology as a predictor for heterotypic development at primary-school age, which has not been investigated before and may add to the knowledge about conditions for heterotypic or homotypic development [e.g., (24)]. Our sample characteristics show a distribution of emotional disorders and behavioral disorders similar to samples from other studies [(25); see also (26)]. Additionally, our analyses examined a disorder cluster labeled *specific early onset disorders*, which probably cover more transient problems in preschool age. The three disorder clusters, ED, BD, and SEO, allow for the examination of cluster comorbidities and cluster specific trajectories. A first finding in this study is that nearly all children at preschool and primary-school age are at high risk of demonstrating a disorder in another disorder cluster (see Table 2 and Figure 1). Note that many studies in the field do not report descriptive details about comorbidity and trajectories, which we consider as a main strength of our study. Detailed information about comorbidities is important (27, 28) to describe, analyze, and interpret heterotypic development [for a broad discussion about comorbidity, see (29); for anxiety see (30, 31)]. In Figure 1 we illustrated the developmental pathways from different starting points, such as children with disorders in the SEO and ED clusters, while the terms “homotypic” and “heterotypic” development covered these different trajectories. A second important sample description is that 50.6% of the children showed homotypic development [in line with (32)] and 44.6% showed heterotypic development, which is in line with findings reported by (1); see also (11). Such knowledge increases the meaning of a mental disorder at preschool age and may emphasize the importance for early diagnosis and intervention (33). The distribution of homotypic and heterotypic development in our sample represents a statistically acceptable situation to examine conditions that influence these percentages.

### Prediction of Homotypic Development

Importantly, our odds ratios in Tables 2, 3 are based on base rates from a clinical sample, which may differ quite substantially from an odds ratios base rate derived from a community sample. First, we interpreted the odds ratios in Table 3 to predict homotypic development. ED and especially BD is, as expected, a predictor of homotypic development. Children with SEO do not show a continuity of the problems, indicating a more transient nature for this class of mental health disorders. Maternal psychopathology seems not to trigger the continuity of emotional problems at preschool age into primary-school age. Contrary to our expectations, we found that the assessment interval is a significant (but small in magnitude) predictor of homotypic development. It is probable that emotional and behavioral problems were temporarily interrupted by an intervention and after months or years, the child will again show the identified problems.

### Prediction of Heterotypic Development

A heterotypic development may be triggered e.g. by emotion-regulation strategies across psychopathology (13). ED seems to represent a negative predictor of heterotypic development, concurring with findings by (7). Moreover, we found that heterotypic development is not predicted by BD, which means that children showing problem behavior in this disorder cluster at preschool age do not tend to generate new (e.g., ED) mental health disorders by primary-school age in line with Aldao et al. (13). This finding seems not support the “failure model” from Capaldi (6); Finsaas et al. (4). Furthermore, we found for our clinical sample that SEO represent not a risk factor to develop new disorders, indicating again the more transient nature for this class of mental health disorders.

Maternal psychopathology, if it remains unimproved at the end of therapy, appears as an exceptionally strong predictive indicator for heterotypic development, with an odds ratio of 6.6 and this finding supports our conceptualization that maternal psychopathology is probably an unspecific stressor for the child that causes additional problem behavior over the years. However, the mechanism by which maternal psychopathology influences heterotypic development (via mother-child-interaction, parenting, social factors) was not examined in this study. The long-term outcome can therefore not be interpreted as an effect related to our Family Day Hospital interventions because approximately half of the sample received a subsequent child-related treatment in the follow-up period. Moreover, maternal psychopathology at discharge is not confounded with subsequent child-related treatment and cannot invalidate maternal psychopathology as a risk factor for a heterotypic development. Additionally it should be noted, that perhaps heterotypic trajectory increased the maternal stress e.g., in case that behavioral problems accompany emotional ones. Finally also uncovered background variables like parental couple separation trigger child and maternal stress (we would like to thank the unknown reviewer for these additional causal relationship).

## Limitations

The sample size limits our statistical power and our probably specific monocentric clinical cohort sample prohibits an overgeneralization of our results to other clinical population. Regardless of the insignificant test of systematic drop-out in our pretesting scores, we cannot rule out that the post-testing score may biased by selection variables such as contact by telephone, change of home address, and so on.

Secondly, our clinical diagnoses at admission were based on clinical records because no clinical interview in German was available. Moreover, the assessment method did not comprise an assessment of developmental disorders at primary-school age. Our analysis did not include further potential risk factors like marital status, number of siblings, and others. Finally, diagnostic assessments at preschool age face several limitations [see (9, 34–36)].

## Ethics Statement

Aktenzeichen 2013-621– f-s Written Consent of participants Ethikkommission der medizinischen Fakultät der WWU Von-Esmarch-Str. 62 48149 Münster.

## Author Contributions

All authors contributed to the final manuscript in conceptual discussions, literature Review, methods and Analysis and IJ especially contributed the Major part.

### Conflict of Interest Statement

The authors declare that the research was conducted in the absence of any commercial or financial relationships that could be construed as a potential conflict of interest.
